# Exploring the dynamics of situational interest in team-based learning in undergraduate medical education

**DOI:** 10.1186/s12909-024-05769-5

**Published:** 2024-07-30

**Authors:** Jerome I. Rotgans, Irene Sterpu, Lotta Herling, Jonas Nordquist, Ganesh Acharya

**Affiliations:** 1 Department of Medicine (Huddinge), Karolinska Institutet, Stockholm, Sweden; 2 Division of Obstetrics and Gynecology, Department of Clinical Sciences, Intervention and Technology (CLINTEC), Karolinska Institutet, Alfred Nobels Allé 8, Stockholm, 141 52 Sweden; 3 Center for Fetal Medicine, Pregnancy Care and Delivery, Karolinska University Hospital, Stockholm, Sweden; 4 Department of Clinical Medicine, UiT The Arctic University of Norway, Tromsø, Norway; 5 Institute for Medical Education Research Rotterdam (iMERR), Erasmus University Medical Center, Rotterdam, The Netherlands

**Keywords:** Team-based learning, Situational interest, Clinical medical education, Knowledge-deprivation hypothesis

## Abstract

**Background:**

Team-based learning (TBL) is a widely recognized instructional approach in medical education blending direct instruction with active-cooperative learning in small groups. While TBL is known to enhance knowledge acquisition, its impact on student motivation, particularly through situational interest, remains underexplored. This study aimed to investigate the development of situational interest across the distinct phases of TBL, focusing on how each phase (individual readiness assurance test; iRAT, team readiness assurance test; tRAT, and application exercise; AE) influences students’ situational interest. The study sought to provide insights into the motivational dynamics underpinning TBL in a medical education setting.

**Methods:**

A total of 88 medical students participated in a TBL session on “Bleeding during Pregnancy.” Situational interest was measured after each TBL phase. A one-way repeated-measures analysis of variance (ANOVA) was conducted to assess the fluctuation of situational interest throughout the session.

**Results:**

The analysis revealed significant variations in situational interest across different TBL stages. There was a significant increase in situational interest following the tRAT (*p* = .001). Post-tRAT, situational interest significantly decreased after the AE (*p* = .007), returning to levels observed at the session’s start. Post hoc correlation analysis suggested a negative association between tRAT performance and situational interest, indicating heightened interest in response to awareness of knowledge gaps during the tRAT.

**Conclusions:**

The findings of this study may challenge the traditional view of TBL, suggesting a more integrated and dynamic interplay between knowledge acquisition and application phases. The results highlight the importance of the AE phase in clinical education and suggest that situational interest is one key driver in the learning process within TBL. Future research should focus on replicating these findings and comparing situational interest development between pre-clinical and clinical student cohorts to further understand the effects of situational interest on TBL in medical education.

## Introduction

Team-based learning (TBL) is an educational approach that blends direct instruction and active-cooperative learning within small groups. The TBL process is typically divided into three stages [1–3]. The initial stage, known as the individual preparation phase [4], involves students independently studying materials such as book chapters, articles, or digital resources provided by their instructors. Following this, the students convene in class to embark on the second stage, the readiness assurance phase. This phase begins with an individual, closed-book knowledge test (also known as the iRAT or individual readiness assurance test) designed to assess the students’ comprehension of the study materials. The same test is then retaken by the students in small groups of 5–7 students, known as the tRAT or team readiness assurance test. During the tRAT, students engage in discussions to reach a consensus on their team answers, after which the correct answers are disclosed [3]. The final stage is the application exercise (AE) phase, where the small groups participate in a series of exercises designed to encourage the application of their newly acquired knowledge.

Since the adaptation of TBL in medical education, studies have investigated how students acquire and consolidate knowledge in TBL [5], how it fosters student accountability [6], improves critical thinking [7], and enhances student learning compared to conventional lectures [8]. While the results indicate that TBL supports learning and enhances student performance, our understanding is still limited in medical education when it comes to how TBL influences and stimulates student motivation to learn. Motivation is a crucial aspect of the learning process, and TBL, with its collaborative and interactive structure, could potentially foster a learning environment that encourages students to actively participate and engage in the learning process, thereby enhancing their motivation to learn.

Research in medical education only recently has begun to explore motivational constructs within the context of TBL. For instance, a study by Jeno et al. (2017) compared the effects of lecture-based and team-based courses on student motivation and learning [9]. The findings revealed that students in team-based courses exhibited higher levels of autonomous motivation and competence compared to those in lecture-based courses. This suggests that the collaborative nature of TBL can foster a more intrinsically motivated learning environment. Additionally, a study conducted by Kim (2017) investigated the impact of TBL on nursing students’ learning attitude, motivation, problem-solving ability, and class participation [10]. The results indicated a significant increase in problem-solving ability, learning attitude, and importantly learning motivation following the implementation of TBL. These studies underscore the potential of TBL to enhance student motivation and learning outcomes, highlighting the need for further investigation into the specific motivational mechanisms that underpin TBL in medical education.

One such mechanism that has the potential to better understand how specific phases in the TBL sequence trigger students’ interest to learn, is the *knowledge-deprivation hypothesis* of situational interest [11]. Unlike broader and more dispositional motivational constructs (e.g., intrinsic motivation, individual interest), situational interest is the interest that is triggered by instructional stimuli, such as problems, puzzles, questions, ambiguous statements, brainteasers, unexpected events, and unfamiliar or surprising situations. According to this theory, when students encounter such an instructional stimulus they do not immediately understand, they attempt to retrieve relevant knowledge from their long-term memory. If this retrieval fails, students experience a knowledge deficit, which triggers situational interest and motivates them to seek out new information to close the knowledge gap, hence the name knowledge-deprivation hypothesis.

Rotgans and Schmidt provided empirical support for the knowledge-deprivation hypothesis of situational interest, predominantly in the secondary school context [12]. They have demonstrated that situational interest is only aroused when students lack knowledge of a topic at hand. Only when students become aware that there is a gap between what they know about a topic and what needs to be known, does situational interest increase. In their view, therefore, aroused situational interest signifies a need for knowledge. However, if the need for knowledge is satisfied, for instance through instruction or self-study, situational interest decreases.

Building on the knowledge-deprivation hypothesis of situational interest, we set out to test this theory in TBL sessions in undergraduate medical education. With its structure of repeated quizzes (i.e., iRAT, tRAT, and AE), TBL may serve as an effective instructional strategy to repeatedly trigger situational interest. We therefore hypothesized that the quizzes in TBL have the potential to make students aware of their knowledge deficits, thereby stimulating situational interest. As students engage in these quizzes, they are likely to recognize the gap between their current understanding and the required knowledge. This recognition, according to the knowledge-deprivation hypothesis, can increase situational interest, signifying a need for knowledge. As students subsequently acquire the necessary knowledge through group discussions and feedback, their situational interest may decrease, indicating satisfaction of their knowledge needs.

Despite the extensive application of TBL in medical education and its proven benefits in fostering knowledge acquisition and critical thinking in preclinical disciplines, there remains a gap in understanding how TBL influences and stimulates student motivation, particularly through situational interest. This study aims to bridge this gap by exploring the development of situational interest across the distinct phases of TBL, thereby providing insights into the motivational dynamics underpinning this increasingly popular instructional approach in medical education.

To achieve this objective, we measured situational interest after each of the distinct TBL phases, in a micro-analytical fashion [13]. Our hypotheses were founded on the knowledge-deprivation hypothesis of situational interest and were as follows:

### Hypothesis 1

Students’ situational interest would be relatively low at the onset of the class, prior to the iRAT, as they would not perceive a significant knowledge gap. Following the iRAT and ensuing feedback, we anticipated a surge in situational interest due to the awareness of knowledge deficiencies. Hypothesis 2: During the tRAT, situational interest would decrease as students engage in collaborative discussions and consultations with content experts, bridging their knowledge gaps. Hypothesis 3: In the AE phase, situational interest would once again be low as students apply and consolidate their understanding in a medical context, with content experts addressing any remaining knowledge gaps.

**Fig. 1:**
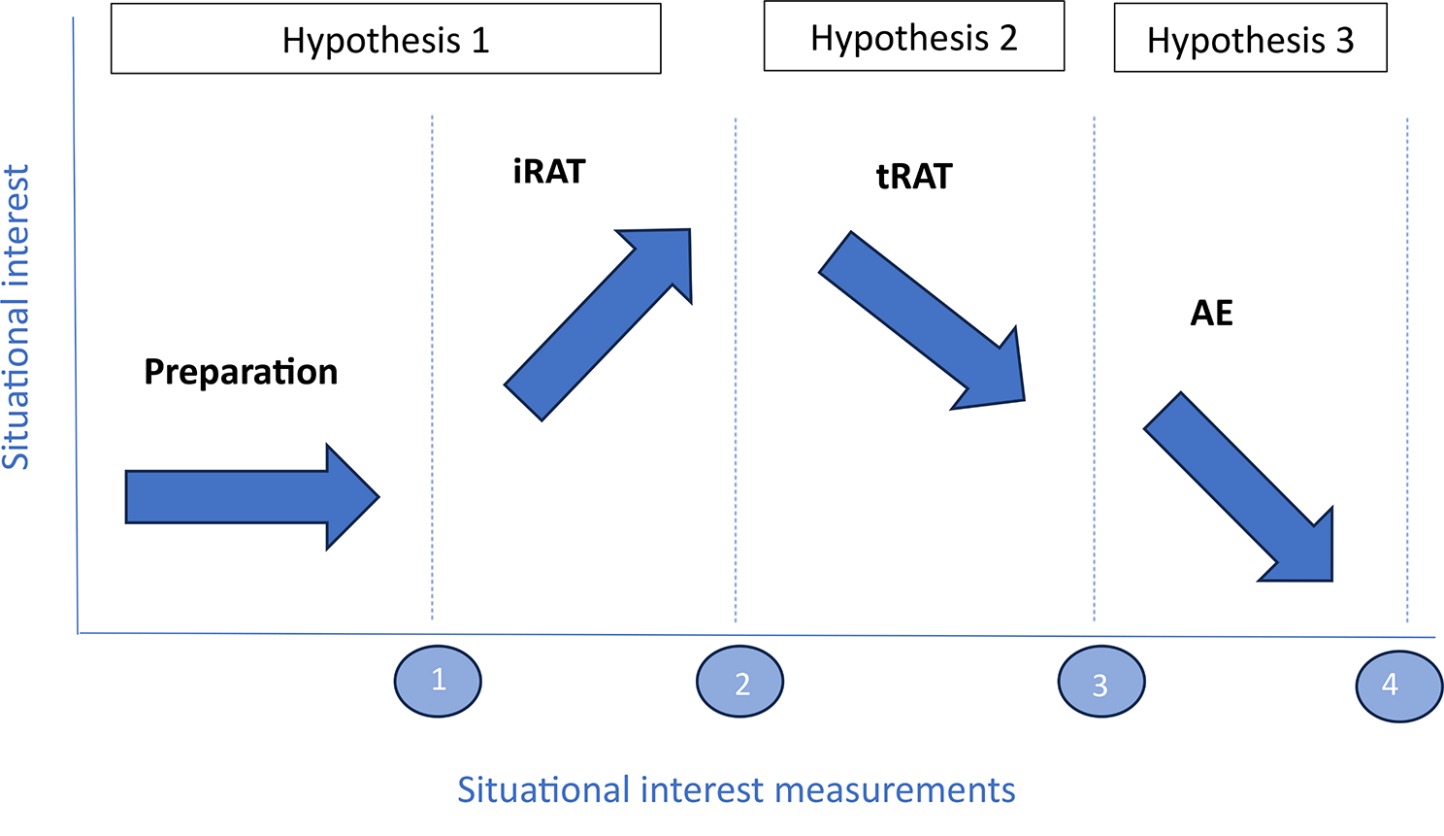
Hypothesis on the situational interest variation during TBL

For a visual overview of the TBL phases, situational interest measurements and hypothesis see Fig. 1.

## Method

### Participants

Out of 122 5th-year medical students who attended the TBL sessions in the Obstetrics and Gynecology course at Karolinska Institutet, Stockholm, Sweden, 88 participated in the study (response rate: 72%). The average age of the aprticipants was 26.55 years (*SD* = 3.63) and there were 59 female and 28 male participants (one student preferred not to respond). The topic of the TBL session was “Bleeding during Pregnancy”. Data were collected during Spring and Autumn 2023. The study protocol was reviewed by the Swedish Ethical Review Authority and granted exempt status on 2022.06.22 (Ref. Dnr: 2022-02891-01). Participation was voluntary and oral information was provided about the study. By choosing to answer the questionnaires, the students gave their informed consent to participate in the study.

### Materials

#### Situational interest measure

Rotgans and Schmidt’s situational interest questionnaire was used in this study, which has shown adequate validity and reliability [11, 14]. The instrument consists of six items (sample items: “*I think this topic is interesting” and “I want to know more about this topic”*) that load on a single latent factor. All items were scored on a 5-point Likert scale: 1 (*not true at all*), 2 (*not true for me*), 3 (*neutral*), 4 (*true for me*), and 5 (*very true for me*). The coefficient *H* was used as a measure of construct reliability and was as follows; situational interest measure 1: *H* = 0.90, situational interest measure 2: *H* = 0.93, situational interest measure 3: *H* = 0.93, and situational interest measure 4: *H* = 0.95. These values suggest high reliability of the measure.

#### Individual readiness assurance test and team readiness assurance test (iRAT/tRAT)

The iRAT and tRAT consisted of 10 multiple-choice questions. An example question:

*A 23-year-old G2P1 (2 pregnancies*,* parity 1) seeks emergency care due to vaginal bleeding and abdominal pain. Her last menstruation was 6 weeks ago. In the emergency department her blood pressure is 100/60*,* her heart rate 100*,* and her Hb is 90. Her pregnancy test is positive. The vaginal ultrasound shows no visible intrauterine pregnancy and large amounts of fluid (suspected blood) in the pouch of Douglas. Which is the most likely diagnosis?*

1. Miscarriage.
2. Cyst rupture.
3. Anovulatory bleeding.
4. Extrauterine pregnancy.

#### Application exercise (AE)

The AE consisted of 7 exercises that guided students in the application of their conceptual knowledge to a medical context. The AE were clinical cases where students were expected to provide very short answers. An example exercise:

*Eva is 22 years old and arrives at the gynecological emergency department due to an acyclical vaginal bleeding. She is on contraceptive pills and thus her cycles are regular. In the emergency room*,* the pregnancy test is positive. The patient´s vital parameters are normal. You conduct a gynecological examination and see some dark brown blood in the vagina. The uterus is normal in size*,* and tender during palpation. No other abnormalities are palpated over the adnexa. An ultrasound scan shows no intrauterine pregnancy*,* and no free fluid in the abdomen. S-hCG is 600 IU/L.*

A. What is your management strategy and how do you inform Eva?
B. *The new S-hCG after 48 h is 900 IU/L. Eva is still without pain and feels fine; she has moderate vaginal bleeding and no visible pregnancy on the ultrasound scan. What do you tell Eva and how do you manage the case?*
C. *2 days later Eva presents at the emergency room with pain on the right side of her abdomen. S-hCG is 1200 IU/L and no intrauterine pregnancy can be seen with ultrasound*,* but a moderate amount of fluid in the pouch of Douglas is now visible. What do you do now?*

The iRAT, tRAT, and AE used in this study are integral components of the regular curriculum and have been in use for a couple of years. These assessments were initially developed by faculty members and subject matter experts to align with the learning objectives of the course. Over the last year, they have undergone periodic reviews and revisions based on student performance data and feedback to ensure their continued relevance and effectiveness.

### Procedure

The TBL session on “Bleeding during pregnancy” was structured sequentially, commencing with a preparation phase of four hours duration before the classroom TBL session. When students came to the session, they were informed about the study. The students were provided with QR-codes and the first situational interest measure was administered online via Google Forms. This was followed by the iRAT lasting 15 min, after which the second situational interest measure was administered. Subsequently, the tRAT was conducted over 25 min, during which the teams responded to the same iRAT questions as a group. This was followed by structured inter-team discussions for a duration of 35 min. After this, the third situational interest measure was administered. Students then progressed to the AE for a 30-minute period, which incorporated additional inter-team discussions lasting another 35 min. The final situational interest measure was then administered after the inter-team discussion.

### Analysis

Responses to negatively stated items were reversed so that for all items the highest scores were indicative of a positive rating. The mean situational interest scores were then calculated for the four administrations and subjected to a one-way repeated-measures analyses of variance (ANOVA) to examine fluctuations in participants’ situational interest during the TBL session. In this study, missing value analysis was employed using the Expectation Maximization (EM) algorithm in SPSS (version 26) to address the issue of non-responses in the questionnaire data. This approach was deemed appropriate given that the proportion of missing values slightly exceeded the 10% threshold, a commonly accepted limit for the application of such techniques in data analysis [15]. For all analyses, the p-value was set to *p* = .05.

## Results

**Fig. 2:**
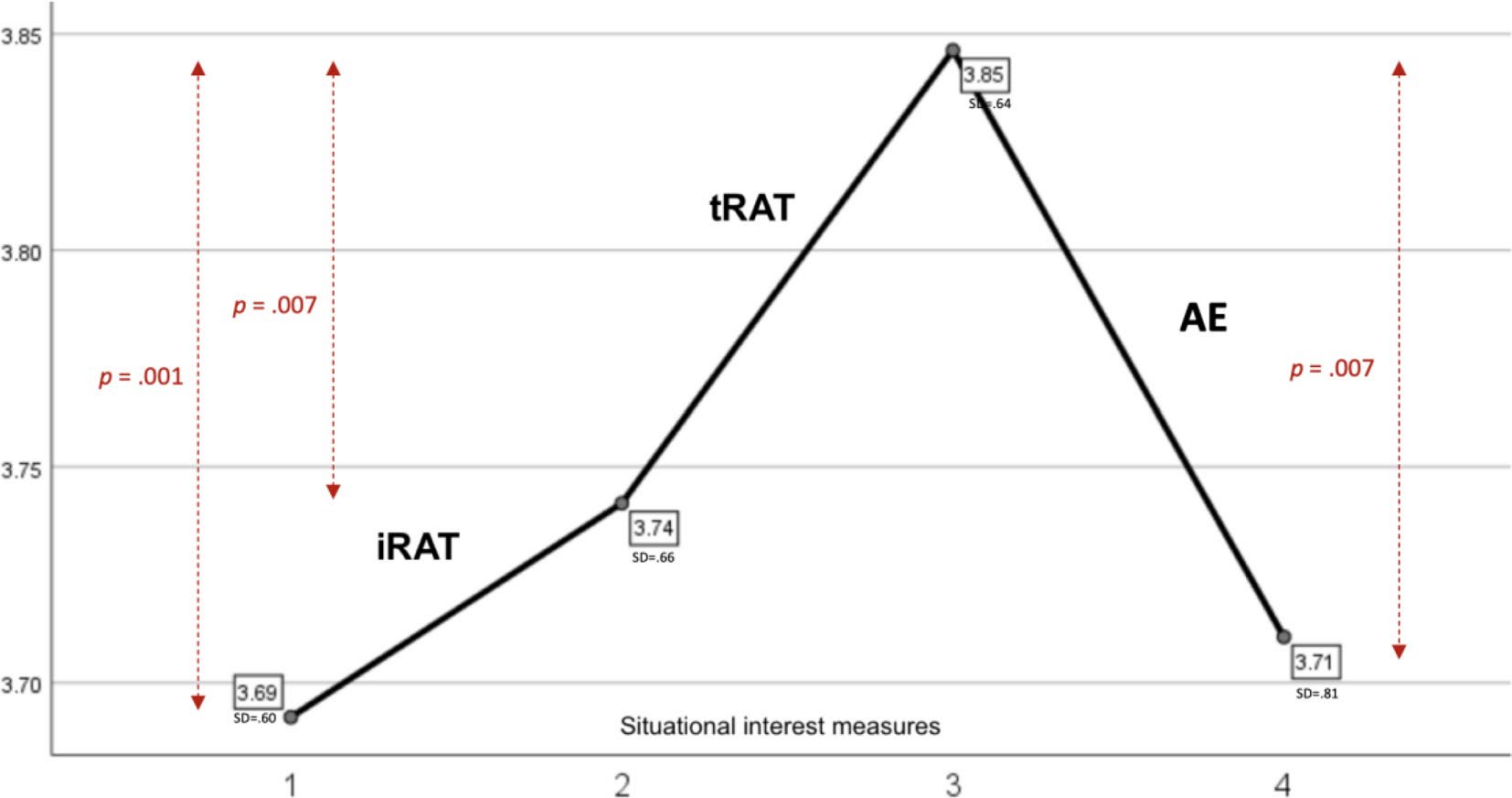
Situational interest development before [1] and after [2] the individual readiness assurance test **(**iRAT), [3] after the team readiness assurance test (tRAT), and [4] after the application exercise (AE). NOTE: Y-axis is from 3.70 to 3.85 on a scale ranging from 1 to 5

The one-way repeated-measures ANOVA revealed a significant effect of time on situational interest, Wilk’s Λ = 0.82, *F* = 6.20, *p* < .001, partial η^2^ = 0.18. Planned pairwise comparisons indicated statistically significant differences in mean scores across three measures of situational interest. Specifically, there was a significant increase in situational interest from Measure 1 (baseline) to Measure 3 (post-tRAT), *p* = .001. A significant increase was observed from Measure 2 (post-iRAT) to Measure 3, *p* = .007. Furthermore, a significant decrease in situational interest was observed from Measure 3 to Measure 4 (post-AE), *p* = .007. The increase between Measure 1 and 2 was not significant, *p* = .19. The same was the case for Measure 1 and 4 (*p* = .79). Finally, the decrease between Measures 2 and 4 was also not statistically significant (*p* = .60). These results are represented in Fig. 2.

## Discussion

The objective of the current study was to investigate the relationship between situational interest and the distinct phases of Team-Based Learning (TBL). We measured situational interest repeatedly during a TBL session, hypothesizing fluctuating levels based on the knowledge-deprivation hypothesis. Specifically, we predicted [1] initially low situational interest before the iRAT, an increase post-iRAT upon realization of knowledge gaps, [2] a decrease during the tRAT as gaps were addressed, and [3] low situational interest during the application exercises (AE) as students applied and consolidated their knowledge (Fig. 1). While our hypotheses were solely based on the knowledge-deprivation hypothesis, it is important to acknowledge at this point that situational interest can be influenced by various other factors, including the quality of instruction, team dynamics, fatigue throughout the session, and the attractiveness of the learning materials.

Despite a modest rise in students’ situational interest following the iRAT, this increment did not reach statistical significance (*p* = .19). Unexpectedly, situational interest exhibited a significant upsurge after the tRAT (*p* = .001). Subsequently, upon completion of the AE, there was a significant decrease in situational interest (*p* = .007), reverting to levels comparable to those observed at the start of the TBL session.

How can these findings be explained? According to the knowledge-deprivation hypothesis, these findings suggest that the tRAT, rather than aiding students in bridging their knowledge gaps, likely heightened their awareness of these gaps, thereby leading to increased levels of situational interest.

To test this alternative hypothesis, we conducted a follow-up correlation analysis between the tRAT scores and situational interest Measure 3. The results showed a negative correlation (*r* = − .19, *p* = .07), albeit not statistically significant, suggesting that teams who performed lower on the tRAT reported higher mean levels of situational interest. No significant correlation was found between the iRAT and situational interest Measure 3 (*r* = .12, *p* = .29). The absence of a significant correlation between the iRAT scores and situational interest Measure 3 further strengthens our suggestion that the observed increase in situational interest after the tRAT is more likely related to the students´ experience of the tRAT itself, rather than other factors such as inadequate preparation for the TBL session.

Moreover, the observed significant decline in situational interest following the AE suggests that students may begin to bridge the knowledge gaps generated during the tRAT during this phase. It is also important to consider that this drop in situational interest could be influenced by other factors beyond knowledge acquisition.

This pattern may underscore the critical role of the AE, particularly in clinical education, as it seems to provide a platform for students to apply and contextualize their theoretical knowledge in a practical clinical setting. There is a likelihood that among these more advanced level (5th year) students, medical knowledge and clinical experience have already been integrated into more efficient cognitive structures, a process known as knowledge encapsulation [16]. The discussions and interactions during the tRAT may therefore have served as a catalyst for heightened situational interest, which in turn, energized and facilitated student learning during the more concrete clinical contexts provided by the application exercises.

### Strengths and limitations

To the best of our knowledge, this study represents an initial attempt to investigate the role of situational interest within the Team-Based Learning (TBL) framework in medical education. However, it is important to acknowledge that our findings are provisional and warrant further verification.

This study has several limitations that should be considered when interpreting the results. Firstly, the small sample size limits the generalizability of our findings. Therefore, a logical progression in this research would be to repeat our study with another larger cohort of medical students in clinical years to replicate our findings. Secondly, the study was conducted at a single institution. Replicating this study across multiple institutions with varying educational environments would help to validate the findings and ensure their broader relevance. Additionally, conducting a parallel study with pre-clinical students would enable a more comparative analysis of situational interest development patterns between pre-clinical and clinical cohorts, thus enriching our understanding of the motivational mechanism that supports the TBL process across different stages of medical education.

The practical relevance of investigating situational interest lies in its potential to enhance student engagement and learning outcomes. Situational interest can be highly dynamic and influenced by multiple factors throughout a TBL session. Understanding how situational interest fluctuates can help educators design more effective instructional strategies to maintain high levels of engagement. While it is beneficial to trigger situational interest throughout the entire session, it is also essential to recognize that fluctuations in interest can signal moments where additional support or adjustment in teaching strategies may be needed (see increased situational interest after the tRAT). Therefore, the goal should be to create a strategic approach that triggers situational interest but also allows for its decrease so that effective knowledge consolidation can occur.

## Conclusions

In conclusion, our findings may suggest that the traditional TBL framework, which typically delineates a clear separation between conceptual knowledge acquisition in the readiness assurance phase and its subsequent application in the AE phase [17–20], might not be as distinct or uniformly applicable as assumed. Although this conclusion is drawn from both situational interest measures and knowledge measures, including the iRAT and tRAT, further research is necessary to verify these findings.

## Data Availability

Data is available and will be provided on demand by irene.sterpu@ki.se.
